# *Staphylococcus epidermidis* Strategies to Avoid Killing by Human Neutrophils

**DOI:** 10.1371/journal.ppat.1001133

**Published:** 2010-10-07

**Authors:** Gordon Y. C. Cheung, Kevin Rigby, Rong Wang, Shu Y. Queck, Kevin R. Braughton, Adeline R. Whitney, Martin Teintze, Frank R. DeLeo, Michael Otto

**Affiliations:** 1 Laboratory of Human Bacterial Pathogenesis, National Institute of Allergy and Infectious Diseases, The National Institutes of Health, Bethesda, Maryland, United States of America; 2 Laboratory of Human Bacterial Pathogenesis, National Institute of Allergy and Infectious Diseases, The National Institutes of Health, Hamilton, Montana, United States of America; 3 Chemistry & Biochemistry Department, Montana State University, Bozeman, Montana, United States of America; Harvard Medical School, United States of America

## Abstract

*Staphylococcus epidermidis* is a leading nosocomial pathogen. In contrast to its more aggressive relative *S. aureus*, it causes chronic rather than acute infections. In highly virulent *S. aureus*, phenol-soluble modulins (PSMs) contribute significantly to immune evasion and aggressive virulence by their strong ability to lyse human neutrophils. Members of the PSM family are also produced by *S. epidermidis*, but their role in immune evasion is not known. Notably, strong cytolytic capacity of *S. epidermidis* PSMs would be at odds with the notion that *S. epidermidis* is a less aggressive pathogen than *S. aureus*, prompting us to examine the biological activities of *S. epidermidis* PSMs. Surprisingly, we found that *S. epidermidis* has the capacity to produce PSMδ, a potent leukocyte toxin, representing the first potent cytolysin to be identified in that pathogen. However, production of strongly cytolytic PSMs was low in *S. epidermidis*, explaining its low cytolytic potency. Interestingly, the different approaches of *S. epidermidis* and *S. aureus* to causing human disease are thus reflected by the adaptation of biological activities within one family of virulence determinants, the PSMs. Nevertheless, *S. epidermidis* has the capacity to evade neutrophil killing, a phenomenon we found is partly mediated by resistance mechanisms to antimicrobial peptides (AMPs), including the protease SepA, which degrades AMPs, and the AMP sensor/resistance regulator, Aps (GraRS). These findings establish a significant function of SepA and Aps in *S. epidermidis* immune evasion and explain in part why *S. epidermidis* may evade elimination by innate host defense despite the lack of cytolytic toxin expression. Our study shows that the strategy of *S. epidermidis* to evade elimination by human neutrophils is characterized by a passive defense approach and provides molecular evidence to support the notion that *S. epidermidis* is a less aggressive pathogen than *S. aureus*.

## Author Summary

*Staphylococcus epidermidis* frequently causes chronic infections, indicating pronounced capacity to evade host defenses. However, *S. epidermidis* is in general much less aggressive than its close relative, *S. aureus*. Here we identify molecular underpinnings of that discrepancy by showing that *S. epidermidis* immune evasion mechanisms are limited to those involving molecules that protect against or eliminate antimicrobial agents secreted by white blood cells, while immune evasion mechanisms of virulent *S. aureus* include the production of destructive toxins. This is especially noteworthy, because we demonstrate here for the first time that *S. epidermidis* has the capacity to produce a toxin with great potential to destroy white blood cells, but keeps its production at a very limited level. Thus, our study shows that two closely related human pathogens have adapted specific molecular mechanisms to evade host defenses, reflecting the unique approach used by each to cause human disease.

## Introduction

*Staphylococcus epidermidis* colonizes the epithelial surfaces of every human being. Furthermore, it is one of the most frequent causes of nosocomial infections. In addition to the abundant prevalence of *S. epidermidis* on the human skin, this high incidence is mainly due to the exceptional capacity of *S. epidermidis* to stick to the surfaces of indwelling medical devices during device insertion and form multilayered agglomerations called biofilms [1], [2].

During infection, *S. epidermidis* is exposed to human innate host defenses, most notably professional phagocytes, among which neutrophils or polymorphonuclear leukocytes (PMNs) play a preeminent role [3]. While the biofilm mode of growth is believed to be broadly protective against host defenses [1], [4], we lack information on specific molecules of *S. epidermidis* that provide resistance to host defense mechanisms. The only *S. epidermidis* molecules known to facilitate evasion of killing by neutrophils are the extracellular polymers poly-N-acetylglucosamine (PNAG, or PIA, polysaccharide intercellular adhesin) and poly-γ-glutamic acid (PGA), which inhibit uptake by neutrophils (phagocytosis) [5], [6]. This is in contrast to *S. aureus*, a more pathogenic relative of *S. epidermidis*, which produces a series of proteins and enzymes dedicated to evade innate and adaptive host defense [7], [8].

Immune evasion of *S. aureus* is due in part to cytolytic toxins, such as α-toxin, γ-toxin, or Panton-Valentine leukocidin, which are proinflammatory and have potential to lyse neutrophils and other leukocytes [9]. In addition, we recently identified a new class of *S. aureus* cytolytic toxins, the phenol-soluble modulins (PSMs). Several PSM peptides have high capacity to attract, stimulate and lyse human neutrophils, and are significant contributors to pathogenesis of *S. aureus* bacteremia and skin infection [10]. PSMα3, in particular, is the most cytolytic *S. aureus* PSM and encoded together with three other PSMs in the *psmα* operon of *S. aureus*. High expression of peptides encoded in the *psmα* operon is mainly responsible for the pronounced potential of hyper-virulent community-associated methicillin-resistant *S. aureus* (CA-MRSA) strains to lyse human neutrophils [10], underpinning the importance of PSMs for neutrophil lysis. In contrast to *S. aureus*, toxins that lyse human leukocytes or other cell types have not been described in *S. epidermidis*.

**Figure 1:**
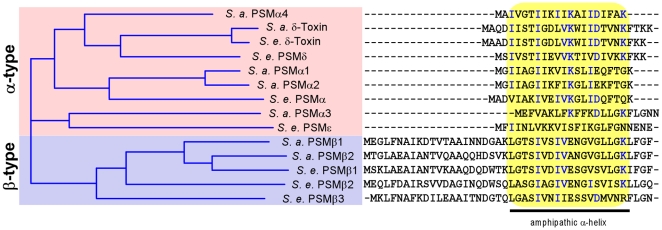
*S. epidermidis* and *S. aureus* PSMs. All known *S. aureus* (*S. a.*) and *S. epidermidis* (*S. e.*) PSMs were aligned by a sequence comparison program (Vector NTI). Similarity on the amino acid level is depicted as a tree on the left. Aligned amino acid sequences are shown at the right, with conserved amino acids shown in blue. All PSMs contain a region with pronounced amphipathy and α-helicity, boxed in yellow.

PSMs are characterized by common physico-chemical properties rather than similarity at the amino acid sequence level (Fig. 1). Identification of PSMs thus requires isolation and characterization by means such as mass spectrometry and Edman degradation. Using these methods, six members of the PSM family have been identified in *S. epidermidis* (Fig. 1) [11], [12], [13], [14], but their biological significance is largely undefined. This is in part due to the fact that in earlier studies, a partially purified extract from *S. epidermidis* containing PSMs was used to measure PSM activities [12], [15], [16], [17]. Therefore, it is possible that proinflammatory activities previously attributed to *S. epidermidis* PSMs were caused by contaminants such as lipopeptides, particularly as similar impurities have frequently led to the misinterpretation of stimulatory effects on innate immune system mechanisms in the past [18]. This emphasizes the need to analyze pure peptides, but pure *S. epidermidis* PSMs and especially cytolytic potencies of *S. epidermidis* PSMs have never been investigated.

After phagocytosis, neutrophils kill bacteria with reactive oxygen species and non-oxygen-dependent processes [19]. Among the latter, antimicrobial peptides (AMPs) such as defensins and cathelicidins are believed to play a crucial role [20]. We have previously found that the secreted *S. epidermidis* protease SepA has considerable capacity to eliminate AMPs by proteolysis [21]. Furthermore, we identified the first Gram-positive AMP sensing system in *S. epidermidis*, *apsRSX* [22]. This system, which has also been named *graRSX* in *S. aureus* [23], [24], regulates a series of AMP resistance mechanisms, including Dlt-dependent D-alanylation of teichoic acids [25], MprF-dependent lysinylation of phospholipids [26], and an AMP exporter called VraFG [24]. However, it is not known whether Aps or SepA confer resistance to killing by neutrophils.

In the present study, we examined the role of *S. epidermidis* PSMs in immune evasion, in particular by determining whether *S. epidermidis* PSMs are cytolytic toward human neutrophils. Furthermore, we analyzed whether the *sepA* and *apsRSX* loci facilitate survival during phagocytic interaction with neutrophils. Our study provides a better understanding of how *S. epidermidis* evades killing by human leukocytes in the susceptible host. Notably, we identified the first potent *S. epidermidis* cytolysin, PSMδ, a member of the α-type PSM family. However, despite the capacity to produce a potent cytolysin, *S. epidermidis* culture supernatants had little or no capacity to lyse neutrophils. In contrast, we show that the SepA protease and the Aps AMP sensor significantly promote resistance of *S. epidermidis* to killing by neutrophils. These findings provide molecular evidence to support the notion that *S. epidermidis*, in strong contrast to virulent *S. aureus*, has a defensive rather than aggressive approach to infection and immune evasion.

## Results

### Cytolytic activity of *S. epidermidis* culture filtrates

To evaluate the relative potency of *S. epidermidis* to kill human neutrophils, we compared culture filtrates of different *S. epidermidis* strains with those of *S. aureus* LAC, a CA-MRSA strain with demonstrated high capacity to lyse neutrophils [10], [27]. We investigated four *S. epidermidis* strains that have been most frequently used in *S. epidermidis* pathogenesis studies: 1457, O47, ATCC12228, and RP62A. ATCC12228 and RP62A represent the two *S. epidermidis* strains for which genome sequence data are available [14], [28]. Furthermore, we included an *agr* mutant of strain 1457, as the *agr* regulatory system is known to strictly regulate PSM production [10], [29], [30].

**Figure 2:**
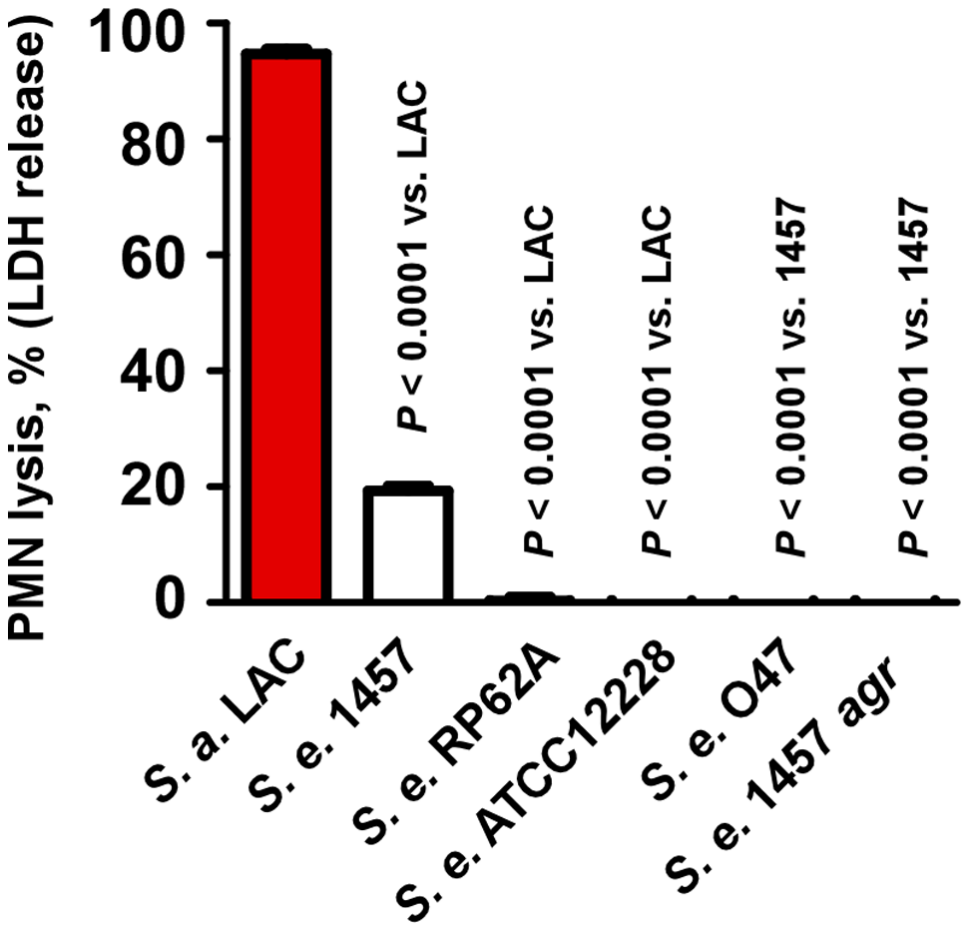
Neutrophil lysis by *S. epidermidis* culture filtrates. Neutrophil (PMN) lysis by undiluted *S. epidermidis* 18-h culture filtrates was determined by measuring release of LDH (incubation time, 1 h). Culture filtrate from *S. aureus* LAC (18-h culture) was used as a comparison.

Culture filtrates of all four *S. epidermidis* strains showed significantly reduced lysis of human neutrophils compared to *S. aureus* LAC (Fig. 2), indicating that as a species *S. epidermidis* has low capacity to lyse neutrophils. Some low-level cytolysis was detected in culture filtrates from strain 1457, but not strains RP62A and ATCC12228. Furthermore, cytolytic capacity of culture filtrates was completely abolished in an *agr* deletion mutant of strain 1457 and in the natural *agr* mutant strain O47 (Fig. 2), in accordance with a potential function of the *agr*-regulated PSMs of *S. epidermidis* in neutrophil lysis.

### Analysis of PSM secondary structure

In vitro studies using *S. aureus* and *S. epidermidis* γ-toxins and *S. epidermidis* PSMδ indicated that PSMs lead to perturbation of synthetic membrane vesicles and likely work by pore formation in the absence of a specific receptor [31], [32], [33]. Presumably, the capacity of PSMs to lyse cells is thus dependent on their physico-chemical features, namely the ability to form amphipathic α-helices, a characteristic property of pore-forming peptides.

**Figure 3:**
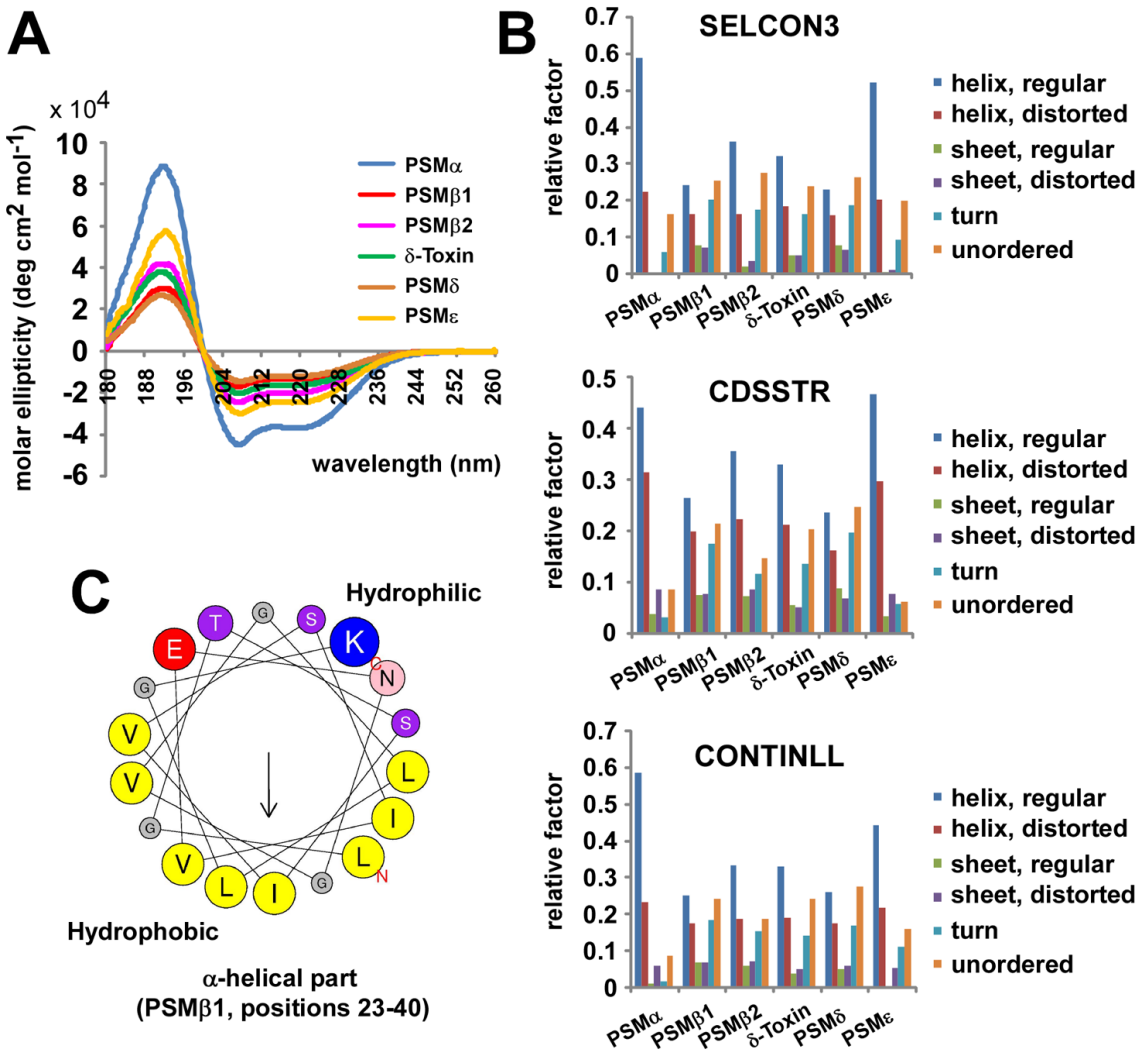
Secondary structure of *S. epidermidis* PSM peptides. Secondary structure of *S. epidermidis* PSM peptides was analyzed by circular dichroism (CD) measurement. (A), molar ellipticity curves; (B) analysis of secondary structure using 3 different algorithms. (C) All PSM peptides have an amphipathic α-helix that encompasses most of the peptide for the shorter α-type and the C-terminal part of the β-type PSMs (shown as example for PSMβ1 by α-helical wheel presentation, http://heliquest.ipmc.cnrs.fr).

To evaluate whether *S. epidermidis* PSMs form amphipathic α-helices, we determined secondary structures of PSM peptides using circular dichroism (Fig. 3A, B). These experiments demonstrated that all *S. epidermidis* PSMs are predominantly α-helical. When PSM sequences were arranged in α-helical wheels, all predicted α-helices showed a distinct hydrophilic opposed to a hydrophobic side, which is characteristic for amphipathic α-helices (shown as an example for PSMβ1 in Fig. 3C). These findings indicate that *S. epidermidis* PSMs have the basic structural requirements for membrane perturbation and pore formation.

### Capacity of *S. epidermidis* PSMs to lyse neutrophils

**Figure 4:**
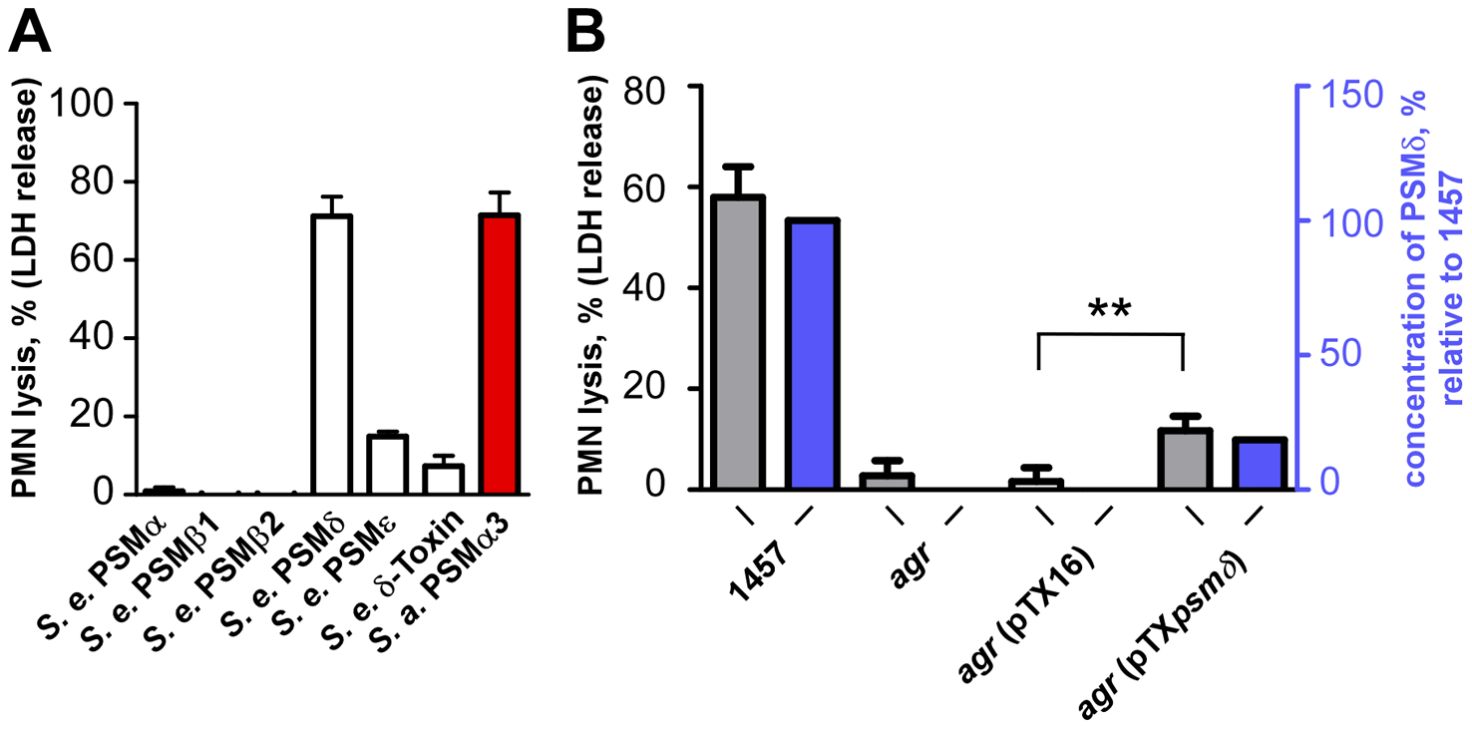
Neutrophil lysis by *S. epidermidis* PSM peptides and culture filtrates of PSMδ-expression strains. (A) Neutrophil (PMN) lysis by synthetic, N-formylated PSM peptides at 10 µg/ml was determined by measuring release of LDH (incubation time, 1 h). PSMα3 (*S. aureus*) was used as a comparison at the same concentration. (B) Neutrophil lysis using supernatants (18-h cultures) of a PSMδ-over-expressing *agr*-negative (lacking intrinsic PSM production) and corresponding control strains (incubation time, 6 h). pTX*psm*δ, pTX construct expressing PSMδ; pTX16, control plasmid. Strains were grown in TSB with 0.5% xylose and 12.5 µg/ml tetracycline. **, p<0.01, paired t-tests. Blue bars, PSMδ concentration in the culture filtrates relative to that in the 1457 wild-type (set to 100%).

To analyze whether *S. epidermidis* PSMs lyse neutrophils, we incubated human neutrophils with pure, synthetic *S. epidermidis* PSMs. Remarkably, one *S. epidermidis* PSM, PSMδ, caused high levels of neutrophil lysis, to an extent comparable to that of the potent *S. aureus* PSMα3 (Fig. 4A). In contrast, *S. epidermidis* δ-toxin, PSMα, and PSMε showed only very limited cytolytic capacity. The β-type PSMs were non-cytolytic toward neutrophils, in keeping with findings achieved for the β-type PSMs of *S. aureus* [10]. These differences indicate that while the formation of amphipathic α-helices is a likely prerequisite for membrane perturbation, further yet unknown structural features determine the degree of cytolytic activity in PSMs. This notion is also supported by our observation that the degree of α-helicity (Fig. 3A) did not correlate with the cytolytic potential of PSMs (Fig. 4A). Of note, PSMδ to our knowledge represents the first potent cytolysin of *S. epidermidis* to be identified. Remarkably, PSMδ is less closely related to *S. aureus* PSMα3 by amino acid sequence comparison than are PSMα1, PSMα2, and *S. epidermidis* PSMε (Fig. 1), underlining the notion that cytolytic properties of PSMs are determined by secondary rather than primary structure.

The strong potency of PSMδ to lyse human neutrophils was confirmed by expression of PSMδ in an *agr*-negative *S. epidermidis* strain that lacks production of PSMs (Fig. 4B). Induction of PSMδ production resulted in a significant increase in the capacity of culture filtrates from the *ag*r-negative strain to lyse human neutrophils (p = 0.0015, *agr* pTX*psm*δ versus *agr* pTX16 control). As we have observed previously [10], [34], plasmid-based expression of PSM peptides often does not result in concentrations of PSMs as high as those found in wild-type culture filtrates, which also was the case for PSMδ. However, the degree of neutrophil lysis exerted by culture filtrates of the PSMδ expression strain (20.1% of that by the wild-type) corresponded very well to PSMδ expression (18.6% of that in the wild-type) (Fig. 4B), highlighting the major contribution PSMδ has to the overall cytolytic capacity of *S. epidermidis*.

### Hemolytic activity of *S. epidermidis* PSMs

**Figure 5:**
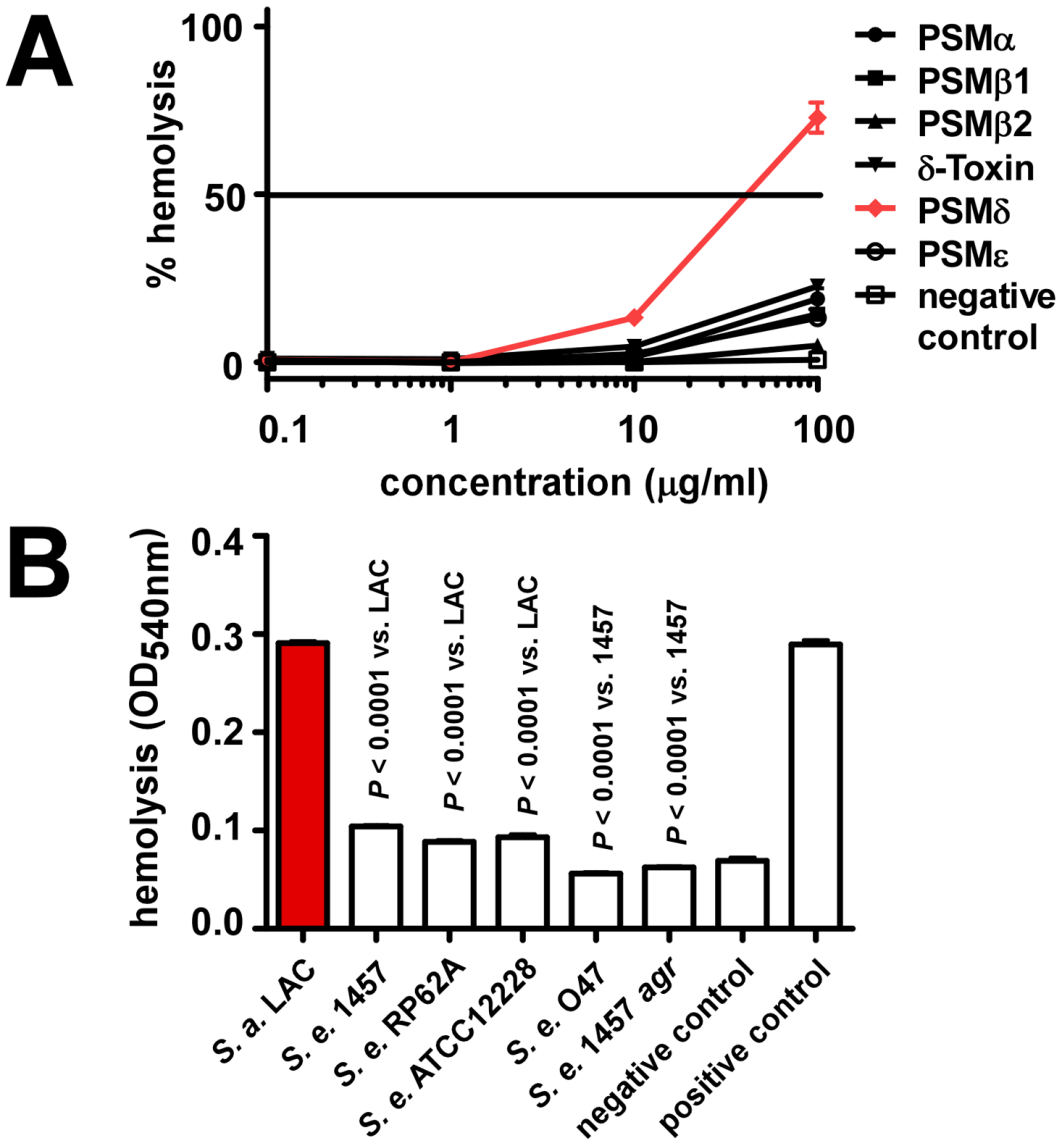
Hemolysis by *S. epidermidis* culture filtrates and PSM peptides. Hemolysis was determined by assays using sheep blood. (A) Hemolysis by synthetic, N-formylated PSMs of *S. epidermidis*. Negative control, DPBS. (B) Hemolysis by *S. epidermidis* culture filtrates (undiluted) and *S. aureus* LAC culture filtrate as comparison. All culture filtrates were from cultures grown for 18 h. Negative control, DPBS; positive control, 1% (v/v) Triton-X100 in DPBS.

We showed previously that *S. aureus* PSMs also lyse cells other than neutrophils, such as monocytes or erythrocytes [10]. To analyze whether lysis of erythrocytes by synthetic PSMs and staphylococcal culture filtrates follows the same pattern as observed using human neutrophils, we tested hemolysis. Results were in very good accordance with those achieved with human neutrophils, inasmuch as only PSMδ showed strong hemolytic activity at a level comparable to that exerted by *S. aureus* PSMα3 (Fig. 5A). Similarly, culture filtrates of *S. epidermidis* strains were much less hemolytic than those of *S. aureus* LAC, with that of *S. epidermidis* 1457 causing slightly higher hemolysis than culture filtrates from the other *S. epidermidis* strains (Fig. 5B), in keeping with the neutrophil lysis findings.

### Production characteristics of PSMs in *S. epidermidis*

**Figure 6:**
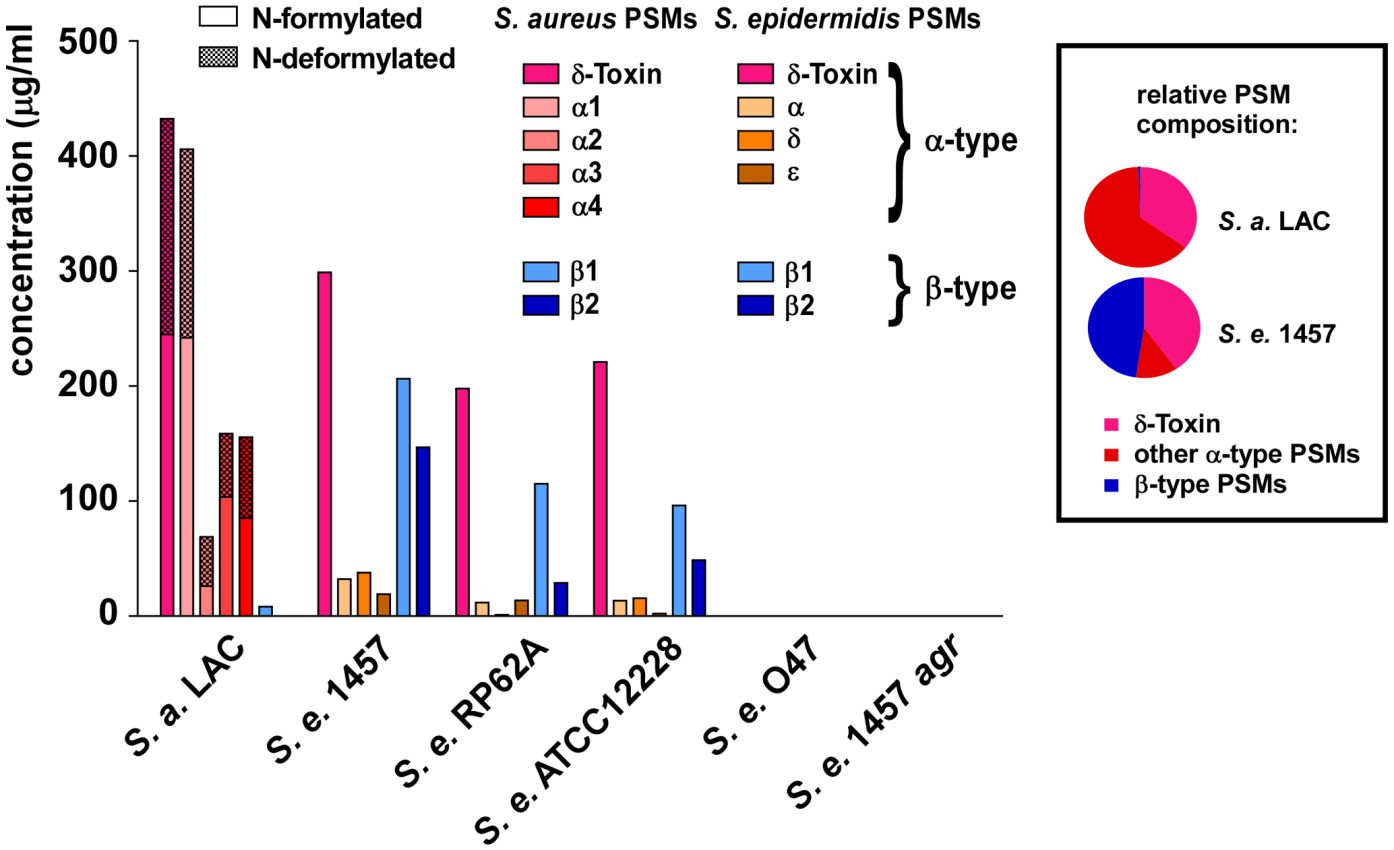
PSM concentrations in *S. epidermidis* culture filtrates. PSM concentrations in 18-h *S. epidermidis* and *S. aureus* LAC culture filtrates were determined by HPLC/MS. Peaks corresponding to N-formylated and deformylated PSM versions were measured separately and the percentage of deformylated peptides is shown as checkered bars. No PSMs were detected in the natural and constructed *agr* mutants (O47, 1457 *agr*). Relative PSM composition (α-type, δ-toxin, β-type) is shown at the right for *S. aureus* LAC and *S. epidermidis* 1457. Relative compositions were similar to that of 1457 in the other *S. epidermidis* strains (except in *agr*-negative O47 and 1457 *agr*).

The finding that *S. epidermidis* PSMδ has considerable cytolytic activity at first appeared to contradict the low cytolytic activity of *S. epidermidis* culture filtrates. Indeed, it was reminiscent of the situation in *S. aureus*, in which the cytolytic potential is also mostly determined by one strongly cytolytic PSM peptide, PSMα3 [10]. However, potential differences in PSM production are not considered in this comparison. Therefore, we next measured PSM production patterns in *S. epidermidis* strains compared to those in *S. aureus*. We found considerable differences in the relative PSM production patterns between *S. aureus* and *S. epidermidis*, while patterns among the different *S. epidermidis* strains were similar (Fig. 6). In addition to the *S. epidermidis* strains that are shown, we analyzed a large *S. epidermidis* strain collection. Results were similar in all strains, except for strains that completely lacked PSM production (data not shown). These PSM-negative strains are likely functionally *agr*-negative, owing to frequently occurring mutations in the *agr* system [35], which includes the *agr*-negative strain O47 [36].

The most noticeable difference between *S. epidermidis* and *S. aureus* was strongly reduced production of α-type PSMs, except δ-toxin, in *S. epidermidis*. In contrast, the non-cytolytic β-type PSMs represented almost half of the total PSM peptide produced in *S. epidermidis*, whereas concentrations of β-type PSMs were extremely low in *S. aureus*. Furthermore, the difference between the production levels of the most cytolytic PSMs in the two species, PSMα3 and PSMδ (∼5∶1), correlated with the degree of overall neutrophil lysis (∼5∶1, *S. aureus* LAC to *S. epidermidis* 1457), underlining that these most potent PSMs predominantly determine cytolytic capacity. Moreover, the notion that any cytolytic activity of *S. epidermidis* is largely determined by production of PSMδ is in accordance with the observed low production of PSMδ and overall low cytolytic activity of all tested *S. epidermidis* strains. Thus, although *S. epidermidis* has the capacity to secrete a potent cytolytic toxin, PSMδ, it limits hemolysis or lysis of neutrophils by keeping production of PSMδ at a low level.

### Deformylation of N-formyl methionine in PSMs

The N-formyl methionine group present at the N-terminus of newly synthesized bacterial proteins is recognized by immune cells as a pathogen-associated molecular pattern (PAMP) [37]. Removal of the N-formyl group by bacterial peptide deformylase thus serves to evade recognition by human innate host defense. N-formylated bacterial proteins commonly are not exported with N-formyl-methionine, as their signal peptides are removed during export. In contrast, PSMs are secreted as the unaltered translation product by a yet unidentified mechanism and thus always carry N-formyl methionine, likely representing a very considerable portion of N-formylated peptides released by staphylococci [10]. In *S. aureus* LAC culture filtrates, about one-half of the total PSM peptide was N-deformylated, which is in good accordance with a previous report on δ-toxin deformylation in another *S. aureus* strain [38]. In remarkable contrast, no significant deformylation was detected in *S. epidermidis* PSMs (Fig. 6). Thus, despite the presence of a peptide deformylase in *S. epidermidis* that is highly homologous to the *S. aureus* enzyme (80% identity on the amino acid level), proteins are not N-deformylated in *S. epidermidis* as efficiently as in *S. aureus*.

### Proinflammatory capacity of *S. epidermidis* culture filtrates and PSMs

**Figure 7:**
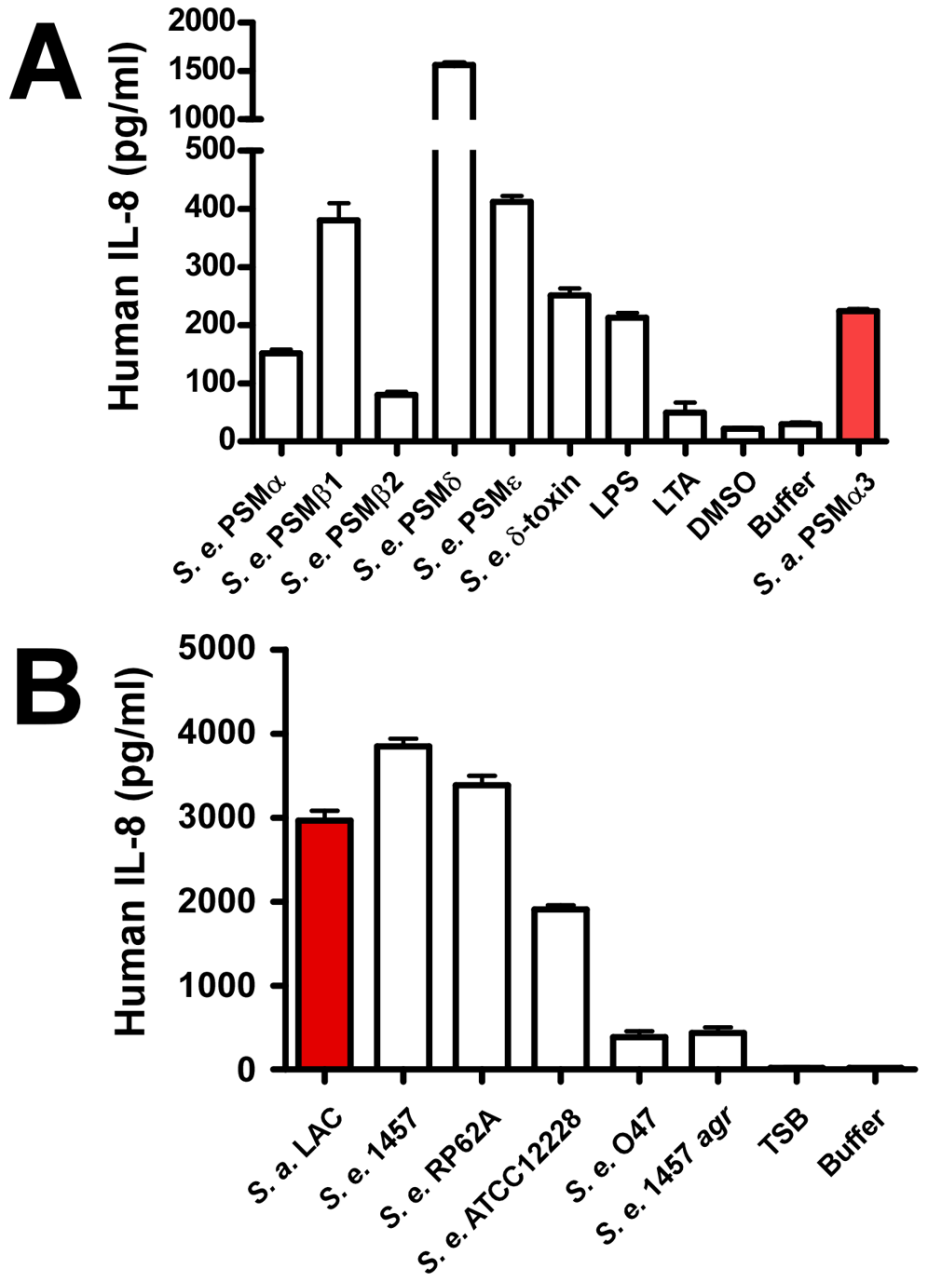
IL-8 release by neutrophils stimulated by *S. epidermidis* PSMs and culture filtrates. PMNs were incubated with synthetic, N-formylated PSMs (10 µg/ml) (A) or 18-h culture filtrates (diluted 1∶100) (B) and release of the cytokine IL-8 was measured by ELISA (for culture filtrates with further 1∶2 dilution). LPS, lipopolysaccharide, 10 ng/ml; LTA, *S. aureus* lipoteichoic acid, 1 µg/ml.

In addition to causing cytolysis, PSMs of *S. aureus* are known to stimulate neutrophil and monocyte chemotaxis, activate neutrophils, and elicit release of the chemokine IL-8 [10]. These proinflammatory capacities of PSMs indicate that the innate immune system recognizes PSMs as PAMPs, which as we recently discovered is achieved by recognition of PSMs by the FPR2/ALX receptor [39]. To determine *S. epidermidis* proinflammatory capacities, we analyzed stimulation of IL-8 release (Fig. 7A). IL-8 is an important chemokine that causes recruitment of neutrophils to the site of infection [40]. PSMδ had very strong capacity to stimulate release of IL-8; but overall, stimulation of IL-8 release did not correlate with the cytolytic capacities of PSMs. Notably, all *S. epidermidis* PSMs to some degree stimulated release of IL-8 despite the lack of cytolytic capacity in several of them. Accordingly, capacities of *S. epidermidis* culture filtrates to stimulate IL-8 release were in the same range as those of *S. aureus* LAC (Fig. 7B). Finally, stimulation of IL-8 release was significantly lower for the *S. epidermidis agr* mutant of strain 1457 compared to the corresponding isogenic wild-type strain, and very low for the natural *agr* mutant strain O47, in keeping with strict regulation of PSMs by *agr* [30]. Thus, while the different PSM production pattern in *S. epidermidis* correlates with considerably reduced neutrophil lysis compared to *S. aureus*, *S. epidermidis* PSMs still appear to be recognized efficiently as PAMPs. These results suggest that PSM cytolytic and proinflammatory capacities are dependent on distinct interactions with host cells.

### SepA protease and Aps AMP sensor/resistance regulator of *S. epidermidis* promote resistance to killing by neutrophils

**Figure 8:**
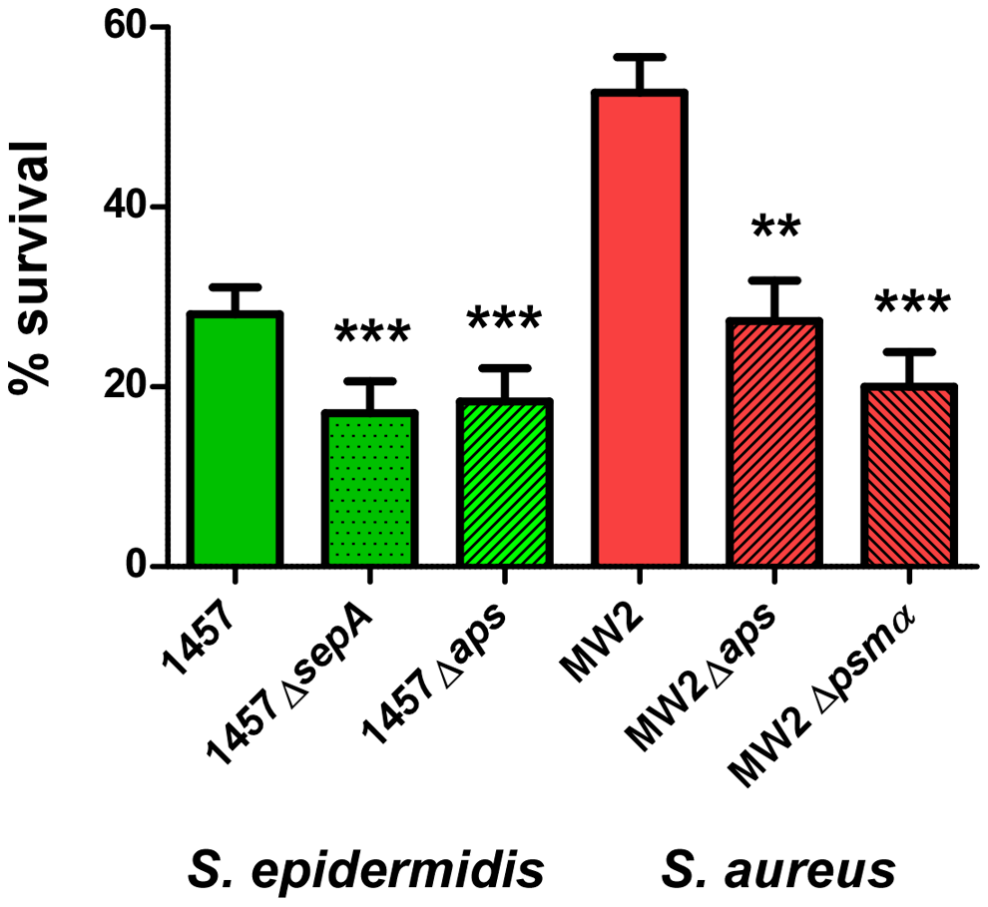
Survival of *aps* and *sepA* deletion mutants in human neutrophils. Survival of *S. epidermidis* 1457 and *S. aureus* MW2 wild-type (wt) and isogenic gene deletion mutants was determined after phagocytic uptake by counting of colony forming units after 60 min incubation. Bacterial cells used for the experiment were harvested at similar points in growth at an OD_600 nm_ of ∼1.5. ***, p<0.001; **, p<0.01 versus the corresponding wild-type sample (1-way ANOVA, Dunnett's post test). Error bars represent SEM.

Our results suggest that *S. epidermidis* does not use PSM cytolytic activity to a significant extent to evade killing by human neutrophils. However, the capacity of *S. epidermidis* to cause chronic infections indicates that *S. epidermidis* has means to inhibit elimination by human professional phagocytes. As an alternative strategy to evade killing by human neutrophils, bacteria may secrete enzymes to destroy – or use mechanisms to decrease – the antimicrobial efficiency of neutrophil bactericidal agents [3]. Among those agents, antimicrobial proteins and peptides likely play an important role in the killing of ingested bacteria [41]. We previously showed that the secreted *S. epidermidis* protease SepA has strong capacity to destroy human AMPs [21]. In addition, we identified a system that we named Aps (for antimicrobial peptide sensor) that senses the presence of human AMPs and coordinates a series of AMP resistance mechanisms in *S. epidermidis* [22] and *S. aureus* [24]. While the mechanistic function of these loci is thus well understood, evidence for a significant role of Aps and SepA in immune evasion using human cells is lacking. Therefore, we investigated whether *S. epidermidis* SepA and *S. epidermidis* and *S. aureus* Aps contribute to survival after uptake by human neutrophils. Isogenic *sepA* and *aps* mutants of *S. epidermidis* 1457 had significantly reduced ability to survive after phagocytic interaction with human neutrophils compared to the wild-type strain (Fig. 8), providing evidence for an important function of the *aps* and *sepA* loci in *S. epidermidis* immune evasion. Similarly, the Aps system had a significant impact on the survival of the *S. aureus* CA-MRSA strain MW2 after phagocytosis. Of note, this effect was comparable to that of the *psmα* locus, which encodes the most important cytolytic PSM peptides of *S. aureus* (Fig. 8B,C). These findings indicate that the Aps AMP-sensing system has an important immune evasion task in both species, while only *S. aureus* makes additional use of cytolytic toxins, such as PSMs, to evade killing by human neutrophils. This discrepancy is reflected by the higher capacity of *S. aureus* to survive interaction with human neutrophils compared to *S. epidermidis* (Fig. 8).

## Discussion

As a commensal organism living on the human skin, *S. epidermidis* commonly has a benign relationship with its host and may even contribute to reducing inflammatory responses [2], [42]. However, *S. epidermidis* may cause infection after breach of the epithelial barrier and entry into the bloodstream, such as through contamination of indwelling medical devices during surgery. Although most *S. epidermidis* infections are only moderately severe and usually chronic, their sheer frequency poses a considerable problem, predominantly in the hospital setting [2], [43]. Despite the immense importance of *S. epidermidis* infections for public health, the interaction of *S. epidermidis* with host defenses is poorly understood. In particular, it has not been investigated in detail if and how *S. epidermidis* resists killing by human neutrophils, which are largely responsible for elimination of invading bacteria. Therefore, we here investigated the interaction of *S. epidermidis* with neutrophils. As direct lysis of neutrophils by bacterial cytolysins is an efficient means to evade killing, we focused our investigation on PSMs as the only *S. epidermidis* gene products with potential cytolytic activity [14], [28].

A major finding of our study was the identification of PSMδ as the first *S. epidermidis* toxin with significant cytolytic capacity. However, despite the cytolytic potential of PSMδ, culture filtrates of *S. epidermidis* strains had very low capacity to lyse human neutrophils. Importantly, according to our findings this phenotypic difference between virulent *S. aureus* and *S. epidermidis* is caused at least in part by a pattern of PSM production in *S. epidermidis* that is shifted, compared to *S. aureus*, to PSMs with lower cytolytic potential. Thus, PSMs in *S. epidermidis* do not contribute significantly to neutrophil lysis, in contrast to many virulent strains of *S. aureus*. Likely, PSMs fulfill other roles in *S. epidermidis* that are yet poorly understood, such as in biofilm development [44] or bacterial interference [33]. The production of PSMs that are not potent cytotoxins would thus ascertain that *S. epidermidis* may cause chronic, biofilm-associated infection without promoting acute, purulent inflammation. This is in keeping with a general strategy of *S. epidermidis* to reside inside the human host in a state of “hiding” from the immune system. Potentially, a similar strategy is pursued by strains of *S. aureus*, such as functionally Agr-negative strains, which are less virulent, cause chronic rather than acute infection, and produce less cytolytic toxins, such as PSMs.

In addition, our study revealed significant contributions of the SepA protease and the Aps AMP sensor/regulator to promoting *S. epidermidis* survival in human neutrophils. Thus, *S. epidermidis* is able to combat important mechanisms that neutrophils use to kill bacteria after phagocytosis. However, together with previous findings on *S. aureus* survival in human neutrophils [27], our data indicate that these mechanisms are not as efficient as leukocyte toxins, underlining the notion that *S. epidermidis* is in general less virulent than *S. aureus* as a result of lower capacity to survive after neutrophil phagocytosis. This is in accordance with a very early study that showed increased survival of “pathogenic” (i.e. coagulase-positive) versus “non-pathogenic” (i.e. coagulase-negative) staphylococci in human leukocytes [45]. Nevertheless, our study shows that - combined with mechanisms preventing neutrophil phagocytosis, such as surface exopolymers and biofilm formation - *S. epidermidis* has a multi-faceted program providing resistance to neutrophil killing, explaining at least in part the capacity of *S. epidermidis* to cause long-lasting infection in the susceptible host. Moreover, as we have shown previously that SepA production is under control of Agr and SarA [21], our findings confirm the notion that global regulatory systems play key roles in *S. epidermidis* immune evasion [46], and are reminiscent of similar functions of Agr and SarA in *S. aureus* [47], [48]. Finally, the observed significant effects of AMP resistance mechanisms on survival in neutrophils underline the importance of non-oxygen-dependent antimicrobial processes of the host.

Collectively, our findings indicate that the molecular mechanisms that *S. epidermidis* uses to evade elimination by innate host defense reflect a passive defense strategy rather than use of aggressive toxins and point to a different major role of PSM production in *S. epidermidis* compared to *S. aureus*.

## Materials and Methods

### Ethics statement

Human neutrophils were obtained from healthy volunteers in accordance with a protocol approved by the Institutional Review Board for Human Subjects, NIAID. Informed written consent was received from human volunteers.

### Bacterial strains and growth conditions

Bacterial strains used in this study were *S. epidermidis* strains 1457 [49], RP62A [14], [50], ATCC12228 [28], O47 [51], isogenic *agr*, *sepA*, and *apsS* deletion mutants of strain 1457 [21], [22], [52], *S. aureus* strains LAC (pulsed-field type USA300) [53] and MW2 (pulsed-field type USA400) [54] and the isogenic *aps* and *psmα* mutants of strain MW2 [24]. LAC and MW2 are virulent community-associated MRSA strains. Strains were grown in tryptic soy broth (TSB). The *psm*δ over-expression plasmid pTX*psm*δ [34] was transformed in *S. epidermidis agr*. Expression of PSMδ by this construct is achieved by adding xylose, which acts on a xylose-inducible promoter in front of the cloned *psm*δ gene [55].

### Peptide synthesis

PSM peptides were synthesized by commercial vendors with an N-terminal formyl methionine residue in each peptide. Peptide sequence fidelity was determined by the Peptide Synthesis Unit of the NIAID. Peptide stock solutions were prepared at 10 mg/ml in DMSO (dimethylsulfoxide); further dilutions were made in water.

### Neutrophil preparation and lysis assays

PMNs were isolated from venous blood of healthy volunteers as described [56]. Lysis of PMNs by synthetic PSMs or clarified *S. aureus* or *S. epidermidis* culture media was determined essentially as described [27], [56]. Synthetic PSMs were added to wells of a 96-well tissue culture plate containing 10^6^ PMNs and plates were incubated at 37°C. After 1 h, PMN lysis was determined by release of lactate dehydrogenase (LDH) (Cytotoxicity Detection Kit, Roche Applied Sciences). Alternatively, *S. aureus* and *S. epidermidis* strains were cultured for 18 h at 37°C in 50 ml TSB (+/− 0.5% xylose) with shaking using a 100 ml flask. Bacteria were removed by centrifugation and culture media were sterilized by filtration and stored at −80°C in aliquots until used. Culture medium was mixed with human PMNs (10^6^) and tested for its ability to cause PMN lysis using incubation times of up to 6 h, as indicated.

### Resistance of *S. epidermidis* and *S. aureus* to killing by human neutrophils

For measurement of *S. epidermidis*/*S. aureus* survival after phagocytic interaction with neutrophils, PMNs (10^6^) in RPMI were combined with ∼10^7^ RPMI-washed bacteria from mid-logarithmic growth phase in 96-well flat-bottom microtiter plates. Plates were centrifuged at 380×*g* for 8 min to synchronize phagocytosis and incubated at 37°C for up to 1 h. At the desired time points, 22 µl of 1% saponin was added, well contents were mixed, and the plates were incubated on ice for 15 min. Surviving bacteria were enumerated. % survival was calculated by comparing the numbers of surviving bacteria to those at t = 0.

### Cytokine production assay

After isolation and washing, neutrophils were resuspended in RPMI 1640 medium (Sigma) supplemented with 10% human serum, 2 mM L-glutamine, 100 U/ml penicillin, 100 µg/ml streptomycin, 2 mM sodium pyruvate, and 10 mM HEPES. Cells were distributed to a 96-well culture plate at 200 µl and 5×10^5^ cells per well. Synthetic PSMs or filtered bacterial culture supernatants were diluted in fresh culture medium (1∶100) and added to the plate at 100 µl/well. Plates were incubated at 37°C in a 5.5% CO_2_ incubator for 5 h. Then, the plate was centrifuged at 1500 rpm for 10 min, and supernatant was harvested from each well. IL-8 was measured in the culture supernatants with commercial ELISA assay kits (R&D systems) according to the manufacturer's instructions. Diluted culture filtrates were further diluted 1∶2 for the ELISA.

### Hemolysis assay

Hemolytic activities of culture filtrates from 18-h cultures of *S. epidermidis* strains or synthetic PSM peptides at different concentrations were determined by incubating samples with sheep red blood cells (2% v/v in Dulbecco's phosphate-buffered saline, DPBS) for 1 h at 37°C as previously described [10]. Assays were performed in triplicate.

### Analysis of PSM production

RP-HPLC/ESI-MS was performed on an Agilent 1100 chromatography system coupled to a Trap SL mass spectrometer using a Zorbax SB-C8 2.3×30 mm column as described [30]. Quantification was based on extracted ion chromatograms using the most abundant peaks of the electrospray ion mass spectra of the respective PSM peptides, with calibration using synthetic peptides, as described [30].

### Circular dichroism spectroscopy

The structures of synthetic PSM peptides were analyzed by CD spectroscopy on a Jasco spectropolarimeter model J-720 instrument. Solutions of PSM peptides, each at 1.0 mg/ml, were prepared in 50% trifluoroethanol. Measurements were performed in triplicate and the resulting scans were averaged, smoothed, and the buffer signal was subtracted. Computation of relative fraction of helix, sheet, turn, and unordered structure, using 3 different algorithms, was performed according to Sreerama and Woody [57].

### Statistical analyses

Statistical analyses were performed with Graph Pad Prism 5 software using t-tests or 1-way ANOVA with Bonferroni or Dunnett post tests, as appropriate.
